# Threat of predation alters aggressive interactions among spotted salamander (*Ambystoma maculatum*) larvae

**DOI:** 10.1002/ece3.3892

**Published:** 2018-02-17

**Authors:** Thomas J. Hossie, Shawn MacFarlane, Amy Clement, Dennis L. Murray

**Affiliations:** ^1^ Department of Biology Trent University Peterborough ON Canada

**Keywords:** amphibian, cannibalism, interference, nonconsumptive predator effects, predator cues, predator–prey

## Abstract

Intraspecific aggression represents a major source of mortality for many animals and is often experienced alongside the threat of predation. The presence of predators can strongly influence ecological systems both directly by consuming prey and indirectly by altering prey behavior or habitat use. As such, the threat of attack by higher level predators may strongly influence agonistic interactions among conspecifics via nonconsumptive (e.g., behaviorally mediated) predator effects. We sought to investigate these interactions experimentally using larval salamanders (*Ambystoma maculatum*) as prey and dragonfly nymphs (*Anax junius*) as predators. Specifically, we quantified salamander behavioral responses to perceived predation risk (PPR) from dragonfly nymphs and determined the degree to which PPR influenced intraspecific aggression (i.e., intraspecific biting and cannibalism) among prey. This included examining the effects of predator exposure on the magnitude of intraspecific biting (i.e., extent of tail damage) and the resulting change in performance (i.e., burst swim speed). Salamander larvae responded to PPR by reducing activity and feeding, but did not increase refuge use. Predator exposure did not significantly influence overall survival; however, the pattern of survival differed among treatments. Larvae exposed to PPR experienced less tail damage from conspecifics, and maximum burst swim speed declined as tail damage became more extensive. Thus, escape ability was more strongly compromised by intraspecific aggression occurring in the absence of predation risk. We conclude that multitrophic indirect effects may importantly modulate intraspecific aggression and should be considered when evaluating the effects of intraspecific competition.

## 1 INTRODUCTION

Aggressive interactions among members of the same species have long interested biologists because of their potential to strongly influence population dynamics (e.g., Arditi & Ginzburg, 2012; Fox, 1975a; Polis, 1981) and individual fitness (e.g., Hokit, Walls, & Blaustein, 1996; Pfennig, 1997; Walls & Roudebush, 1991). Aggressive interference competition benefits attackers by displacing competitors from high‐quality habitats and by reducing the vigor and viability of their competitors through injury (Semlitsch & Reichling, 1989; Wildy, Chivers, Kiesecker, & Blaustein, 2001). Conspecific aggression may also extend to cannibalism where attackers benefit both directly from the associated energetic gains and indirectly by reducing intraspecific competition (Crump, 1992; Fox, 1975a; Polis, 1981). Indeed, the demographic effects of both interference (Arditi & Ginzburg, 2012) and cannibalism (Benoît, McCauley, & Post, 1998; Wissinger, Whiteman, Denoël, Mumford, & Aubee, 2010) can be significant. Investigations into the factors which influence rates of cannibalism in natural systems have focused primarily on the direct effects of density, food availability, and heterogeneity in size or age class (e.g., Fox, 1975a,b; Polis, 1981; Walls, 1998; Wildy et al., 2001). Yet, in addition to the danger posed by conspecifics, many of these animals also experience predation from heterospecific sources, and the threat of attack by predators from a higher trophic level may strongly influence agonistic interactions including cannibalism (Kishida et al., 2011; Rudolf, 2008). A growing body of work highlights the importance of nonconsumptive predator effects in natural systems (Preisser, Bolnick, & Benard, 2005; Suraci, Clinchy, Dill, Roberts, & Zanette, 2016; Zanette, White, Allen, & Clinchy, 2011), and we stand to gain a more comprehensive understanding of intraspecific conflict by characterizing interactions across trophic levels while uncovering the behavioral mechanisms involved.

Given that high conspecific density increases rates of intraspecific aggression and cannibalism (Fox, 1975a; Semlitsch & Reichling, 1989; Wildy et al., 2001), it follows that lethal predation should reduce the rates of such antagonistic interactions among prey by reducing the density of conspecifics. The nonconsumptive effects of predation risk on rates of intraspecific aggression and cannibalism are, however, less clear. For example, predator‐induced reductions in activity and foraging behavior should also reduce the frequency of encounters with aggressive or cannibalistic conspecifics, but congregation in areas that provide refuge from predators might increase time spent in close proximity to hostile competitors or cannibals. Similarly, increased vigilance might reduce both predation and cannibalism, while reductions to active foraging combined with mobilization of energy in preparation for escape could generate hunger that increases the propensity to attack conspecifics viewed as competitors or as prey items. Although the complexity of natural systems often renders it difficult to forecast the outcome of such interactions, the effect of plastic anti‐predator responses on the rate of cannibalism, for example, should generally depend on whether such responses reduce or enhance: (1) vulnerability of conspecifics to cannibals; or (2) propensity of cannibals to attack conspecifics (Rudolf, 2008). Controlled experimental work is therefore well‐suited and necessary to elucidate the mechanisms which underlie these multitrophic interactions.

Spotted salamander (*Ambystoma maculatum*) larvae inhabit vernal ponds where they are vulnerable to predation both by conspecifics (Brodman, 1999, 2004; Brodman & Jaskula, 2002; Walls, 1998; Walls & Jaeger, 1987) and aquatic invertebrate predators (e.g., *Anax* dragonfly nymphs) (Anderson & Petranka, 2003; Petranka, 1998; Yurewicz, 2004). Spotted salamander larvae also exhibit interaspefic biting reflecting a mixture of interference competition and attempted canibalism, with the latter increasing in frequency as size discrepancies widen (Walls, 1998). Conspecific attacks among *Ambystoma* larvae result in damage to the tail, gills, or limbs (Semlitsch & Reichling, 1989; Walls & Jaeger, 1987; Wildy et al., 2001), and the number of injured larvae is correlated with the extent of cannibalism (Semlitsch & Reichling, 1989). Larvae with tail damage tend to be smaller (Petranka, 1989) and may experience reductions in competitive ability or survival relative to uninjured larvae if, for example, tail damage increases susceptibility to infection (e.g., Walls & Jaeger, 1987). Tail damage from conspecifics could also increase susceptibility to subsequent cannibalism or predation through compromised swimming ability or reduced vigor. Interestingly, Semlitsch and Reichling (1989) demonstrated that conspecific aggression among salamander larvae (i.e., tail damage) was lower in simulated drying environments compared to environments with constant water levels, indicating that factors other than density and food availability can strongly shape agonistic interactions between conspecific salamanders. Salamander larvae from many species perceive and respond to predation risk by sensing chemical cues in the water (e.g., Davis & Gabor, 2015; Sih & Kats, 1994). The larvae of spotted salamanders in particular respond to chemical cues from *Anax* dragonfly nymphs by reducing activity and altering tail shape (Shaffery & Relyea, 2015; Yurewicz, 2004), changes that could also affect intraspecific interactions among larvae. Thus, while salamander larvae clearly face threats posed both by aggressive conspecifics and by heterospecific predators, it remains unclear whether responding to predators might influence lethal and sublethal aggressive interactions among conspecifics.

In this study, we sought to: (1) quantify behavioral responses of spotted salamander larvae to perceived predation risk (PPR) from dragonfly nymphs; (2) determine the degree to which intraspecific aggression (i.e., intraspecific biting and cannibalism) was influenced by exposure to PPR; and (3) examine the effect of predator exposure on the magnitude of sublethal intraspecific aggression (i.e., extent of tail damage from intraspecific biting) and the resulting change in performance (i.e., burst swim speed). Specifically, we used a split‐brood design exposing half of the larvae to PPR from dragonfly nymphs and the other half to a control (sham) treatment, then quantified activity, feeding, refuge use, tail damage, burst swim speed, and survival. We predicted that prey responses to predator cues would reduce the extent of agonistic interactions among conspecifics, leading to reductions in cannibalism and tail damage.

## 2 MATERIALS AND METHODS

Fifteen spotted salamander (*A. maculatum*) egg masses were collected in May 2014 near Buckhorn, Ontario, Canada (44°34′17″ N, 78°19′15″ W). To allow us to track kinship of hatching larvae, egg masses were housed individually in plastic bins filled with aged (>24 hr) ozonated river water. Once hatched, salamander larvae were housed in 2 L of aged ozonated river water. Eggs and hatchling larvae were reared for 1 month (from date of collection) prior to the start of the experiment, during which they experienced a 12:12‐h photoperiod, were fed brine shrimp nauplii ad libitum, and were housed in ozonated river water. Ten broods were chosen for our study, and remaining broods were used to maintain predators and generate chemical cues for our experiment. Larvae were weighed 1 day prior to the start of the experiment, and mean larval mass was roughly equivalent among broods (*F*_9,124_ = 1.111, *p* = .36; Table S1). Half of the larvae from each of the 10 broods were then randomly assigned to either a treatment or control tank. Specifically, there were 20 tanks in total, each housing between 5 and 9 larvae with the tanks from corresponding broods possessing equal numbers of larvae (±1 larva; see Table S1). Each tank (15 × 30 × 20 cm) was filled with 2 L of aged ozonated river water, continuously aerated, and given a green plastic enclosure (i.e., a refuge) that provided cover over 33% of the tank area. These larval densities enable us to examine the influence of predator cues on rates of intraspecific aggression and are within the range used in previous work examining intraspecific interactions among *Ambystoma* larvae (e.g., Brodman, 1999; Walls, 1998; Walls & Roudebush, 1991). Natural hatchling densities up to 257.7/m^2^ have been reported for *A. maculatum*, and up to 349.9/m^2^ in other *Ambystoma* species (Cortwright, 1988). We acknowledge that the densities we employed may have been somewhat higher than is typically observed in nature; however, we sought to understand cannibalism and interference, processes which limit or regulate abundance, and are acutely observable at high densities. Moreover, our experiment sought to evaluate broad ecological questions related to trophic interactions and their consequences, rather than precisely recreate natural conditions. A 12‐12‐h photoperiod was used for the duration of the experiment. All procedures performed herein involving animals were reviewed and approved through the institutional review process of Trent University's Animal Care Committee.

Salamander larvae were fed brine shrimp (*Artemia franciscana*) nauplii twice daily. Nauplii were hatched from cysts within solutions of 25 ppm sodium chloride in water. These solutions were stirred in an attempt to homogenize the distribution the nauplii, and 0.5‐ml aliquots of this solution were examined with a magnifying glass to estimate nauplii concentration. Using this estimate, we calculated the volume of *Artemia* solution necessary to provide ~1,000 *Artemia* nauplii per salamander. The nauplii were then filtered and added to fresh ozonated river water. Next, we enumerated the total number of salamander larvae in each tank and a volume of this nauplii solution was added to each tank corresponding to the number of larvae. Ninety percent water changes were performed 1 hr after morning feedings on days when treatments were administered.

Five dragonfly nymphs (*Anax junius*) were individually housed outside of experimental tanks in containers with 500 ml ozonated river water. Predator cues used to manipulate PPR were generated by feeding two salamander larvae to each nymph 2 hr prior to the behavior trials. The water from all five nymph containers was pooled into a separate single container, and 100 ml of aliquots was added to each of the predator treatment tanks. Control tanks instead received 100 ml aged ozonated river water to control for disturbance. Treatments were administered three times per week (i.e., Monday, Wednesday, and Friday), concurrent with afternoon feeding and at least 5 hr after morning water changes had taken place.

Twenty minutes after treatments were administered, we conducted behavioral assays. Specifically, we conducted 30‐s scans to determine the number of larvae feeding (i.e., discrete lunges toward prey items) and the number active (i.e., movement of any kind) for each tank. Number of active larvae included larvae that were feeding because such movements similarly increase detectability to dragonfly nymphs. We also quantified refuge use by counting the number visible and calculating the difference between known total and total visible. Because the total number in each tank differed through time, we converted all behavioral measures to proportions for our analyses. We used linear mixed models to assess the effect of PPR on proportion active, proportion feeding, proportion using refuge, and proportion alive (i.e., survival). In all cases we set tank, nested within brood, as a random effect. To meet the assumptions of our tests, proportion feeding was square root transformed and proportion using refuge was arcsine‐square root transformed. Analyses were conducted in R (R Development Core Team, 2015) using the library nlme (Pinheiro, Bates, DebRoy, Sarkar & R Development Core Team, 2015).

At 2 weeks from the start of the experiment, we weighed and photographed (lateral view) the salamander larvae to assess tail damage. Once photographed, each individual was placed in a water‐filled track under a top‐mounted DSLR camera where we recorded three burst swim events, each initiated by touching the larva's tail with the tip of a pipette. Videos were recorded at 30 frames per second. To extract salamander shape from the photos, we used tpsDig2 (Rohlf, 2006) to digitize 19 landmarks on each photograph. These landmark data were read into CoordGen7a (Sheets, 2011a) where we conducted a Procrustes transformation to eliminate effects of size, rotation, and translation on shape (Zelditch, Swiderski, Sheets, & Fink, 2004). Using the Procrustes transformed landmark data, we then conducted a principal component analysis (PCA) using PCAGen7a (Sheets, 2011b) to generate orthogonal variables that describe and quantify the variation in shape. Principal components (PCs) which described more than 10% of the variation in shape and laid to the left of the “elbow” in the scree plot were considered important. We examined the effect of predator exposure, mass, and their interaction on each of the significant PCs using linear mixed models with tank, nested within brood, as a random effect.

Burst swim videos were converted into image stacks. Dayton, Saenz, Baum, Langerhans, and DeWitt (2005) suggested that the 0.2 s following tactile stimulus is likely the critical time to escape an attack by sit‐and‐wait predators like *A. junius* dragonfly nymphs. We therefore measured the distance traveled in the 0.2 s (i.e., six frames) immediately after the tactile stimulus for each of the three burst swim responses, by extracting the coordinates using the *Manual Tracking* function in Fiji (Schindelin et al., 2012), and calculating Euclidean distance between the points. Distance traveled was converted to millimeters by multiplying this distance by the scaling factor and then divided by 0.2 to obtain speeds in mm/s. From these values, we determined the maximum speed of each individual salamander larvae. In order to examine the effects of treatment and size on shape, as well as the effect of shape and size on maximum swim speed, we conducted structural equation modeling on standardized data (i.e., centered and scaled) using the lavaan package in R (Rosseel, 2012).

We examined differences in mean larval mass per tank at 2 and 4 weeks from the start of the experiment by conducting paired t‐tests (i.e., paired by egg brood); because of especially high rates of cannibalism in two tanks, the second *t*‐test was conducted on eight tank pairs.

## 3 RESULTS

**Figure 1:**
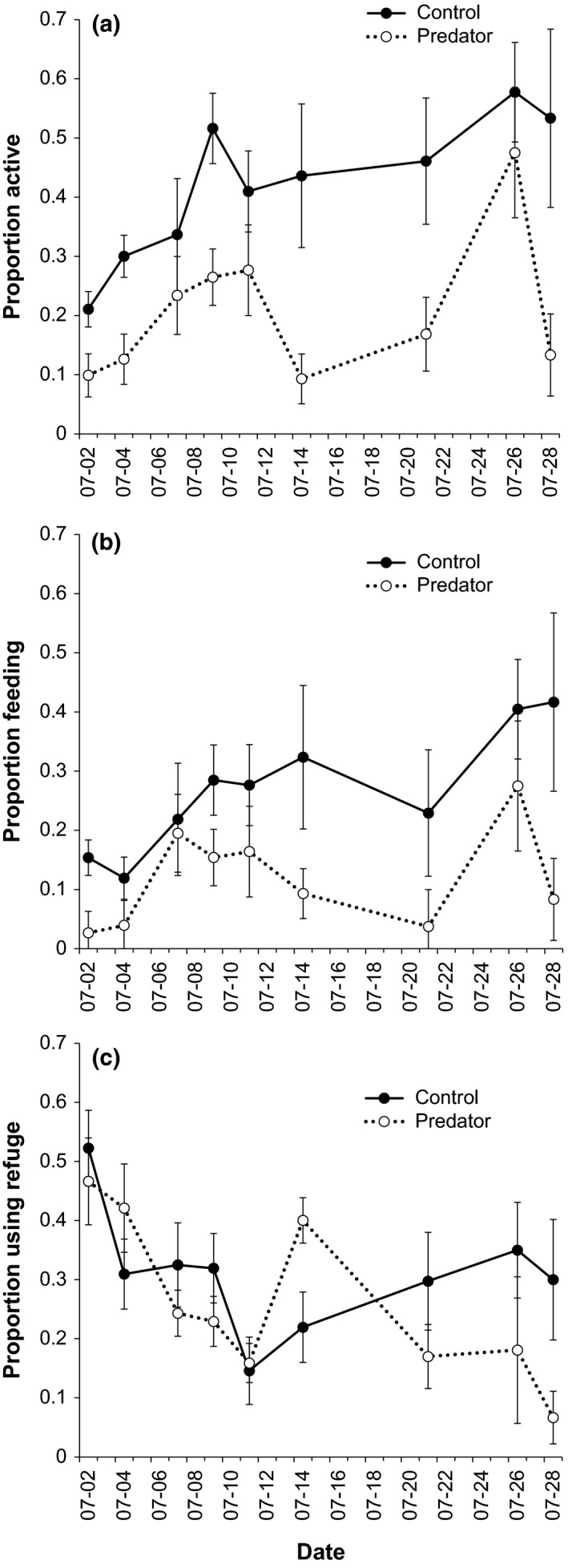
Spotted salamander (*Ambystoma maculatum*) larvae behavioral response to the addition of chemical cues from dragonfly nymphs fed salamander larvae over the course of 26 days. (a) Proportion of larvae active, (b) proportion of larvae feeding, and (c) proportion of larvae using the refuge. Data are reported as tank‐level mean ± *SE*

Exposure to PPR significantly reduced the proportion of salamander larvae that were active (mean ± *SE*: control = 0.42 ± 0.031, predator = 0.21 ± 0.024; *F*_1,9_ = 33.052, *p *<* *.001, Figure 1). The proportion of larvae that were active increased slightly, but significantly, over time (*F*_1,158_ = 13.673, *p *<* *.001, Figure 1), but the effect of PPR did not change over time (*F*_1,158_ = 2.077, *p* = .15). The proportion of salamander larvae feeding was also reduced by exposure to PPR (control = 0.27 ± 0.027, predator = 0.12 ± 0.022; *F*_1,9_ = 27.686, *p* = .005, Figure 1). The proportion of larvae feeding did not change over time (*F*_1,158_ = 1.312, *p* = .25), nor did the effect of PPR on proportion feeding change over time (*F*_1,158_ = 1.141, *p* = .29). PPR did not elicit a change in proportion of larvae using refuge habitat (control = 0.31 ± 0.025, predator = 0.26 ± 0.025; *F*_1,9_ = 0.715, *p* = .42, Figure 1), but there was a slight decrease in the proportion of larvae using refuge over time (*F*_1,158_ = 16.430, *p* = .001). There was no significant interaction between time and presence of predator cues on refuge use (*F*_1,158_ = 3.052, *p* = .083).

**Figure 2:**
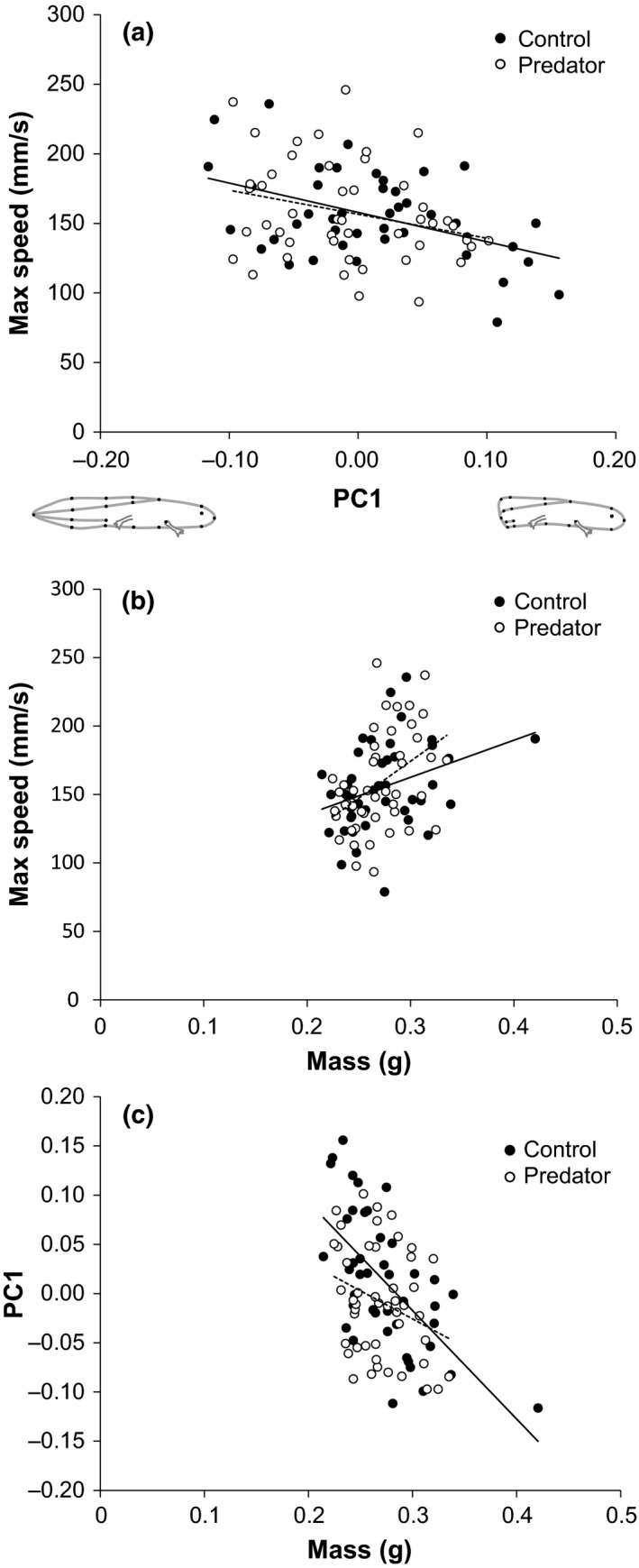
Relationships between body shape, size, and performance in control and predator‐exposed spotted salamander larvae 2 weeks from start of the experiment. (a) Relationship between the extent of tail damage and maximum burst swim speed (mm/s). (b) Relationship between body mass (g) and maximum burst swim speed (mm/s). (c) Relationship between mass (g) and the extent of tail damage. In all panels, solid lines indicate the fitted linear model for control larvae, and dashed lines indicate the fitted linear model for larvae exposed to predator cues

After 2 weeks, 86.3% (44/51) of the remaining larvae in the control treatment had evidence of tail damage from conspecifics, whereas only 70.9% (39/55) of the larvae in predator treatment had damaged tails. Our assessment of shape produced two significant principal components, which explained 63.0% and 13.5% of the variation in shape, respectively (PC3 explained only 7.6%). PC1 represented the extent of tail damage with a larger value indicating more damage (Figure S1, Figure 2), and PC2 represented a slight change in body depth with a larger value indicating a deeper body (see Figure S1). The amount of tail damage (as measured by PC1) decreased with larger body mass (*F*_1,68_ = 41.538, *p *<* *.001), and decreased with exposure to PPR (*F*_1,9_ = 6.170, *p* = .035), but was unaffected by the mass × treatment interaction (*F*_1,68_ = 1.367, *p* = .25). The lower mass of larvae with tail damage was greater than the mass of the missing tissue; larvae with ~50% tail loss were approximately 26.8% lighter than larvae with no tail damage. Body depth (PC2) increased with larger body mass (*F*_1,68_ = 5.204, *p* = .026), but was unaffected by the predator treatment (*F*_1,9_ = 1.703, *p* = .22) or the mass × treatment interaction (*F*_1,68_ = 0.12, *p* = .73).

**Figure 3:**
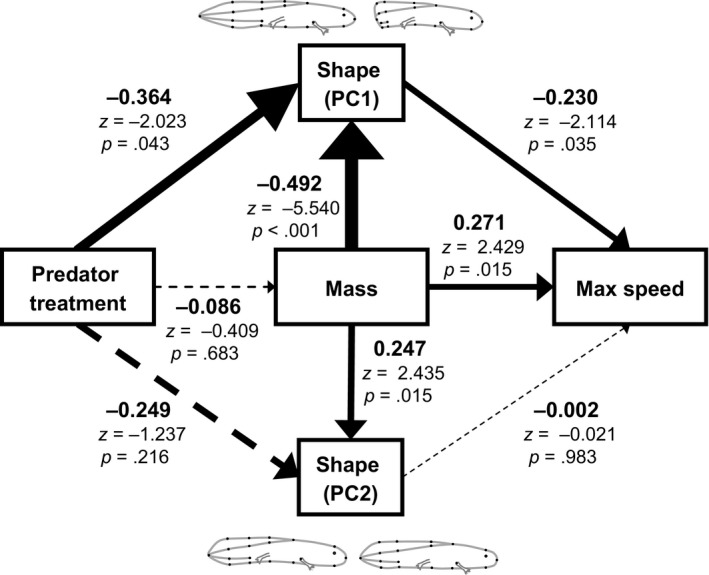
Results from a structural equation model describing the factors that influence body shape, mass, and burst swim speed in larval spotted salamanders. Arrow width is proportional to the size of the effect, and solid arrows indicate significant effects

**Figure 4:**
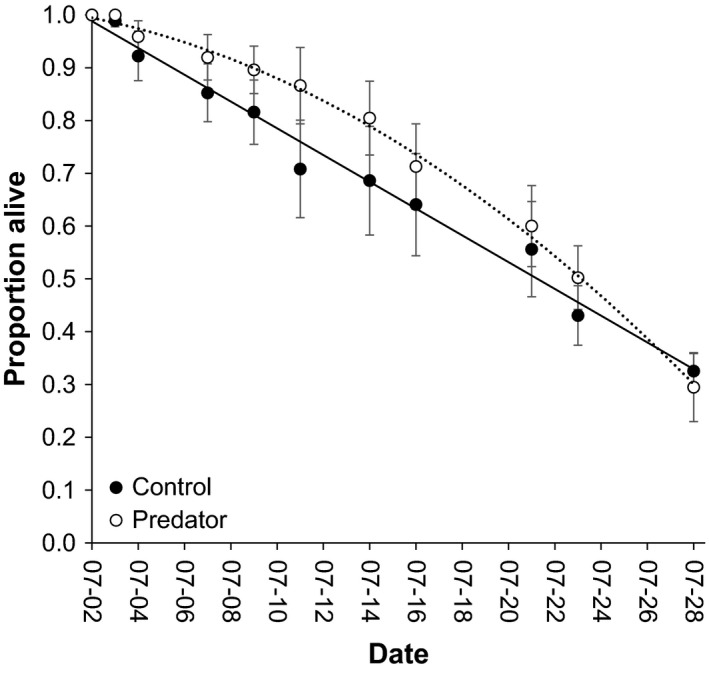
The proportion of spotted salamander larvae alive in experimental tanks treated with control (i.e., water) or chemical uses from dragonfly nymphs fed larval salamanders over the course of a 26‐day experiment. The solid line indicates the line of best fit for control larvae, and the dotted line indicates the curve of best fit for predator‐exposed larvae. Data are reported as tank‐level mean ± *SE*

Structural equation modeling revealed that maximum burst speed decreased as tail damage became more extensive (PC1: *z* = −2.114, *p* = .035; Figures 2a and 3) and increased with body mass (*z* = −2.023, *p* = .043, Figures 2b and 3), but was not affected by body depth (PC2: z = −0.021, *p* = .98; Figure 3). Body mass negatively influenced the extent of tail damage (PC1: *z* = −5.540, *p *<* *.001, Figures 2c and 4) and was positively related to body depth (PC2: *z* = 2.435, *p* = .015; Figure 3). Importantly, predator exposure reduced the extent of tail damage (PC1: *z* = −2.023, *p* = .043), but did not affect body mass (*z* = −0.409, *p* = .68) or body depth (PC2: *z* = −1.237, *p* = .22; Figure 3).

The proportion of salamanders alive declined significantly over the course of the experiment (*F*_1,198_ = 540.288, *p *<* *.001), but was not influenced by the presence of predator cues (*F*_1,9_ = 0.934, *p* = .36) or an interaction between the presence of cues and time (*F*_1,198_ = 0.022, *p* = .88, Figure 4). Interestingly, a linear model best described the survival of control larvae (adjusted *R* ^2^ = .984), and a second order polynomial was not a significantly better fit for the survival of control larvae (adjusted *R* ^2^ = .983, Likelihood Ratio Test: χ^2^ = 0.23 *p* = .63, *df* = 1). In contrast, a second‐order polynomial was a significantly better fit than a linear model for the survival of predator‐exposed larvae (adjusted *R* ^2^: linear = .955, polynomial = 0.995; LRT: χ^2^ = 25.99 *p *<* *.001, *df* = 1). The within‐tank average mass of larvae between treatments did not differ significantly at week 2 (mean ± *SE* = 0.053 ± 0.0072 g; *t* = 0.742, *df* = 9, *p* = .48) or week 4 (0.076 ± 0.0144 g; *t* = −0.578, *df* = 7, *p* = .58). The datasets analyzed during the current study are available from the corresponding author on reasonable request.

## 4 DISCUSSION

Herein we experimentally investigated how lethal and sublethal intraspecific aggression in spotted salamander larvae was influenced by exposure to PPR from dragonfly nymphs. In accordance with previous work, salamander larvae responded to PPR by reducing activity and feeding, but did not increase their use of refuge habitat (Shaffery & Relyea, 2015; Yurewicz, 2004). Thus, conspecific encounter rate was reduced by PPR through reductions in movement, opposed to increasing as a result of predator‐induced shifts in microhabitat use (e.g., aggregation in refuge habitat). Consistent with this, larvae exposed to PPR experienced less tail damage from conspecifics. Exposure to PPR also indirectly influenced maximum burst swim speed which declined as tail damage became more extensive. Larger larvae sustained less tail damage; however, predator treatment did not appear to influence body mass. While we did not detect a significant difference in survival between predator exposed and control larvae, the pattern of survival differed among treatments suggesting that fear of predators may have subtle but important impacts on the rate of cannibalism.

We attribute the reduction in sublethal intraspecific aggression (i.e., tail damage) to the reduced encounter rate between aggressive conspecifics and potential victims resulting from predator‐induced reductions in activity and foraging (Figure 1a,b). Importantly, predator‐induced changes to aggression among conspecifics can result from the nonmutually exclusive mechanisms of: (1) behavioral changes in the potential victims that reduce vulnerability to conspecific attacks; or (2) behavioral changes in the potential aggressors that moderate conspecific attack rates. Unfortunately, we are unable to disentangle the relative importance of these two mechanisms here. Both activity and feeding reductions are well recognized means for amphibian larvae to reduce detectability and encounter rate with predators (e.g., Dijk, Laurila, Orizaola, & Johansson, 2016; Skelly, 1994) and should similarly reduce a potential victim's probability of encountering an aggressive conspecifics. Yet, it remains equally possible that aggressive or cannibalistic individuals moderated their search behavior or attack rate upon exposure to predator cues to minimize their own exposure to predators (e.g., Rudolf, 2008).

Amphibian larvae are well‐known for their ability to alter tail shape in response to PPR (Hossie, Landolt, & Murray, 2017; McIntyre, Baldwin, & Flecker, 2004; Relyea, 2004). In our experiment exposure to predator cues primarily altered the tail shape of salamander larvae not through plasticity, but instead by modifying the interactions among conspecifics (i.e., intraspecific biting). Specifically, larvae exposed to PPR experienced (and inflicted) significantly less tail damage from conspecifics. The amount of tail damage had a significant effect on burst swim speed, and by inference, on escape ability. Therefore, while larvae exposed to PPR from predatory dragonfly nymphs possessed a tail shape which would facilitate rapid escape from natural enemies, escape ability was compromised in the unexposed control treatment by intraspecific aggression (but see Wilbur & Semlitsch, 1990). Critically, the effect of conspecific damage on tail shape, and subsequent performance, may often outweigh the relatively weak plastic predator‐induced modifications to tail shape observed in *A. maculatum* larvae (e.g., see: Yurewicz, 2004; Shaffery & Relyea, 2015).

Salamander body size was significantly related to the amount of tail damage (Figure 2c); however, it remains difficult to determine the direction of causality in this relationship. Petranka (1989) similarly observed that tail‐damaged individuals were smaller and suggested that loss of tail tissue compromised growth; however, Wilbur and Semlitsch (1990) found that tail damage had little effect on growth or development in anuran tadpoles. Alternatively, an association between tail damage and small body size might also be the result of small larvae experiencing greater risk of injury by conspecifics compared to large larvae. Two lines of evidence support such an explanation in our system: (1) smaller larvae have reduced escape ability (e.g., slower burst swim speed; Figure 2b); and (2) cannibalism (and associated sub‐lethal damage to conspecifics) in *Ambystoma* salamander larvae and other taxa tends to be perpetrated by larger individuals and directed toward smaller individuals (e.g., Nyman, Wilkinson, & Hutcherson, 1993; Smith, 1990; Walls, 1998).

The significant effect of exposure to PPR on sublethal intraspecific aggression outlined above was not matched with a significant difference in survival between treatments. That said, we did observe a distinct shift in pattern of survival (Figure 4). Petranka (1989) observed similar variation in the survival curves of *A. opacum* larvae during whole‐pond manipulations. Clearly, our understanding of larval recruitment patterns stands to benefit from additional empirical work that identifies the factors which determine the pattern of survival. In strict terms, our experiment was too short in duration to rigorously characterize the survivorship curve; however, our results suggest that fear of predators may have subtle but important indirect impacts on survival in cannibalistic taxa. Delaying our experiment for 1 month following hatching could have influenced our ability to detect differences in cannibalism; however, larvae typically remain similar in size in the weeks immediately following hatching which should minimize cannibalism during this time. We also note that sufficient size variation among larvae was present upon initiation of our experiment for cannibalism to occur given that it was directly observed on numerous occasions. Instead, failure to observe differences in cannibalism between treatments may be, in part, a result of having housed salamander larvae with members from the same egg mass (i.e., kin). For example, cannibalistic spadefoot toad tadpoles nip at conspecifics, and subsequently consume nonsiblings, but release siblings unharmed (Pfennig, Reeve, & Sherman, 1993; but see also Walls & Blaustein, 1995). Whether exposure to PPR differently affects rates of cannibalism in kin versus nonkin groups remains a potentially fruitful avenue for future theoretical and empirical study. Overall our work indicates that fear of predators can modulate intraspecific aggression and should be considered when evaluating the effects of intraspecific competition.

## CONFLICT OF INTEREST

None declared.

## AUTHOR CONTRIBUTIONS

T.J.H., S.M., A.C., and D.L.M each contributed to the conception, design, data interpretation, and writing of this manuscript. T.J.H., S.M., and A.C. implemented the experiments, collected data, and conducted analyses. All authors gave final approval of this version to be published.
