# Supplementary material for: Threat of predation alters aggressive interactions among spotted salamander (Ambystoma maculatum) larvae
Source: Ecol Evol. 2018 Feb 17;8(6):3131–8. doi: 10.1002/ece3.3892 (PMC5869354; doi:10.1002/ece3.3892)
Supplement: Supplementary file 1 [file ECE3-8-3131-s001.pdf]

Table S1: The average mass (mg) and standard deviation of larvae from 10 spotted salamander egg broods (A-J) at the initiation of our experiment. Larvae were divided evenly between control and predator treatment groups, the number of larvae per tank is provided.

**Egg brood**	**A**	**B**	**C**	**D**	**E**	**F**	**G**	**H**	**I**	**J**	**Total**
Control	5	9	9	6	9	8	9	9	7	8	79
Predator	5	9	9	7	8	7	8	9	8	8	79
$\bar{x}_{mass}$ (mg)	44.6	53.7	44.7	42.5	41.4	54.5	72.9	46.3	49.0	50.1	50.2
σ_{mass} (mg)	8.9	10.2	11.5	7.6	9.6	4.2	110	6.8	7.3	6.1	35.7

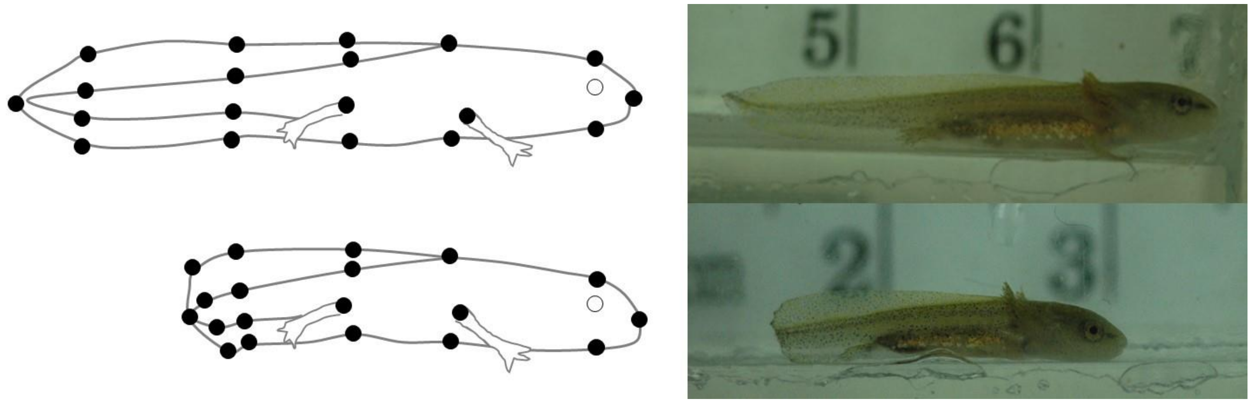

Figure S1: Position of landmarks and the morphological change represented by PC1. On the right are the individual animals corresponding to the shape deformation depicted on the left. Specifically, the top animal has a PC1 score of approximately -0.12, and the animal on the bottom has a score of 0.16. Note that the depicted shape deformation on the left represents the shape change described by PC1 after having removed the independent variation described by other principal components (e.g., PC2).

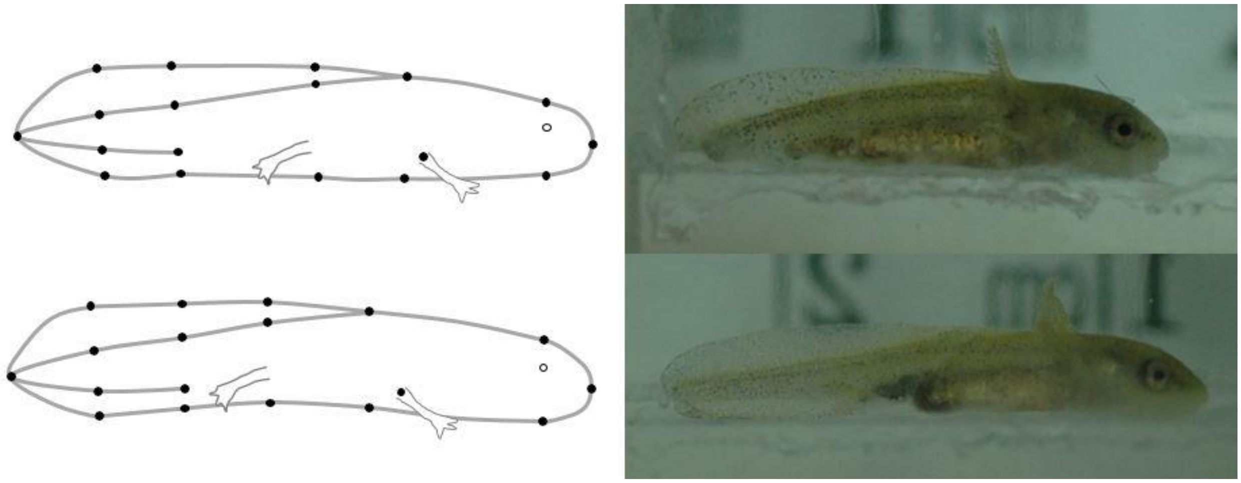

Figure S2: Position of landmarks and the morphological change represented by PC2. On the right are the individual animals corresponding to the shape deformation depicted on the left. Specifically, the top animal has a PC2 score of approximately 0.06, and the animal on the bottom has a score of -0.06. Note that the depicted shape deformation on the left represents the shape change described by PC2 after having removed the independent variation described by other principal components (e.g., PC1).
